# Immune mechanisms of granuloma formation in sarcoidosis and tuberculosis

**DOI:** 10.1172/JCI175264

**Published:** 2024-01-02

**Authors:** Praveen Weeratunga, David R. Moller, Ling-Pei Ho

**Affiliations:** 1 MRC Translational Immunology Discovery Unit, Weatherall Institute of Molecular Medicine, University of Oxford, Oxford, United Kingdom.; 2 John Hopkins University, Baltimore, Maryland, USA.

## Abstract

Sarcoidosis is a complex immune-mediated disease characterized by clusters of immune cells called granulomas. Despite major steps in understanding the cause of this disease, many questions remain. In this Review, we perform a mechanistic interrogation of the immune activities that contribute to granuloma formation in sarcoidosis and compare these processes with its closest mimic, tuberculosis, highlighting shared and divergent immune activities. We examine how *Mycobacterium tuberculosis* is sensed by the immune system; how the granuloma is initiated, formed, and perpetuated in tuberculosis compared with sarcoidosis; and the role of major innate and adaptive immune cells in shaping these processes. Finally, we draw these findings together around several recent high-resolution studies of the granuloma in situ that utilized the latest advances in single-cell technology combined with spatial methods to analyze plausible disease mechanisms. We conclude with an overall view of granuloma formation in sarcoidosis.

## Introduction

Sarcoidosis is a complex immune-mediated granulomatous disease with a prevalence of up to 1:10,000 in some populations (1). Nearly any organ can be involved, but 90% of patients show lung disease on CT scan (2). Clinically, a hallmark of the disease is its varied patterns of clinical presentations and disease course. Many patients recover, even without treatment, whereas others develop chronic granulomatous inflammation, and some develop fibrosis. Some patients are refractory to treatment, while others have self-limiting disease despite a high volume of abnormalities. Sarcoidosis can be classified in different ways, such as by type of onset, disease course, and organ involvement. In those who are symptom free, sarcoidosis is discovered incidentally, usually when the lung is imaged for other reasons. For patients with Löfgren syndrome, the disease is acute, with fever, ankle arthritis, and/or erythema nodosum or uveitis as well as bilateral hilar lymphadenopathy on chest radiograph. These patients tend to have a better prognosis, with 80% of cases resolving within 2 years. In most patients, the onset of symptoms is gradual. The disease course of sarcoidosis includes self-limiting, chronic but stable, or chronic and progressive disease (2).

Genetic association studies, including GWAS, have identified genes and loci that likely contribute synergistically to disease susceptibility and severity (3–42) (Supplemental Table 1; supplemental material available online with this article; https://doi.org/10.1172/JCI175264DS1). The immune profile in sarcoidosis is dominated by Th1 cell overactivity. A variety of antigens have been implicated, but it is generally accepted that there is no unifying etiological cause (2).

The clinical features and etiological conundrums have been collated in several comprehensive reviews (2, 43–46). Here, we intend to review the disease mechanisms in sarcoidosis from the immunological perspective. Other studies have compared immunological profiles in blood and bronchoalveolar lavage (BAL) between sarcoidosis and tuberculosis (TB) (47–50). We take a different approach by focusing primarily on the sarcoidosis granuloma in tissue, considering the granuloma as the driver of disease immunopathology. We interrogate the immune activities contributing to granuloma formation in sarcoidosis and compare these to TB, its closest mimic clinically and histopathologically. We take advantage of the detailed studies in TB and its known inciting antigens and highlight shared and divergent immune activities.

In each section we explore TB first (as the etiology is known), then sarcoidosis. We examine how *Mycobacterium tuberculosis* is sensed by the immune system; how the granuloma is initiated, formed, and perpetuated in both diseases; and the role of major innate and adaptive immune cells in these processes. Studies in TB around these topics far exceed those in sarcoidosis, and they include various animal models, routes of infection, and mycobacterial species. Overall, studies in zebrafish tend to provide information for the earliest stages of granuloma formation, particularly around the role of innate immune cells. Studies using nonhuman primates (NHPs) are highlighted in this Review, and we have generally concentrated on primary pulmonary TB lesions in human lungs and not the postprimary disease (as discussed by Hunter et al., ref. 51). We draw findings from these studies together around several recent high-resolution studies of the granuloma in situ that utilized the latest advances in single-cell technology to analyze plausible disease mechanisms. We conclude with an overall view of granuloma formation in sarcoidosis.

## Granuloma in TB and sarcoidosis: composition and structure

A granuloma is an organized immune entity, comprising macrophages, lymphocytes, and, to a lesser extent, monocytes, neutrophils, and fibroblasts. It is a reaction to an intracellular pathogen or substance that macrophages have failed to destroy. Granulomas can be formed by the innate immune system alone without contribution from adaptive immune cells (52, 53). However, for both TB and sarcoidosis, CD4^^+^^ T lymphocytes are integral to the granulomatous structure. The main cellular component of the granuloma is the macrophage, which develops into two specialized structures: epithelioid cells and multinucleated giant cells (MGCs). In epithelioid cells, macrophages acquire epithelial features such that adjacent cells become linked by interdigitated cell membranes and adherens junctions. This probably represents an attempt by the host to wall off the inciting agent for destruction while simultaneously protecting the host from collateral cellular injury. Most antigens that cause epithelioid transformation are infectious in nature, but there are clear exceptions, e.g., berylliosis, which has classical epithelioid granulomas. Both epithelioid cells and MGCs are found together in TB and sarcoidosis, though epithelioid granulomas are more prevalent in sarcoidosis (54). MGCs may confer greater phagocytosis capabilities due to their larger size and abundant lysosomes. No definitive specific antigens are known to trigger formation of MGCs or epithelioid granulomas.

Central necrosis or caseation of the granuloma is found frequently in TB and other pathogen-driven granulomas but not usually in sarcoidosis granulomas. The cause of necrosis is not entirely understood, but it is clear that *M*. *tuberculosis* thrives in this necrotic material and uses it to disseminate its progeny (55–57). Necrosis has been linked to both under- and overproduction of TNF-α (58–62). TNF deficit results in overwhelming bacterial overgrowth in macrophages, causing necrosis, whereas excess TNF induces apoptosis of macrophages through the production of reactive oxygen species (55, 59, 62). These studies in zebrafish embryos examined the innate and early immune response in the initial stages of granuloma formation but not the chronic phase. Another potential cause for central necrosis is the sheer number of apoptosing, infected macrophages that overwhelm the clearance capacity of fresh macrophages (63, 64). Necrosis in TB granulomas is distinct from fibrinoid necrosis (where fibrin is deposited due to increased vascular leakage) that can occur in sarcoidosis. This crucial difference in morphology between TB and sarcoidosis granuloma suggests that a live, metabolically active process of organism growth is at the center of necrosis in TB but not sarcoidosis granuloma.

It is well established that a spectrum of TB granuloma types are found within a single host, with varying levels of organization and cellular composition. Each granuloma displays independent trajectories that are probably determined by differential killing of *M*. *tuberculosis* after the onset of the adaptive immune response (56, 65–67). *M*. *tuberculosis* burden within individual granulomas tends to be highest in early formed lesions and decreases as the immune response matures, even in individuals who ultimately develop active TB (67, 68). Sarcoidosis granulomas within any individual patient also show significant variability (54). The reasons for this variability are poorly understood, but they could be caused by the variable types of immune cells surrounding the granuloma (69).

## Initiation of granuloma

In TB, macrophages are the first cells to encounter *M*. *tuberculosis* in the lungs, though neutrophils and monocytes can also recognize and be infected by *M*. *tuberculosis*. Many parts of the microbe can act as pathogen-associated molecular patterns (PAMPs), including cell wall components, DNA and cytosolic proteins (ESAT-6 and CFP10), superoxide dismutase A (SOD A), and heat shock proteins (Mtb-hsp and mKatG) (70, 71). These antigenic components are recognized by TLRs (mainly TLR2, -4, and -9), Nod-like receptors, C-type lectin receptors, complement receptors, Fc receptors, GPI-anchored membrane receptors (e.g., CD14), and scavenger receptors (e.g., MARCO and CD36). Sensing of these PAMPs triggers a cascade of events in macrophages, like lysosomal *M*. *tuberculosis* uptake, phagosomal maturation and acidification, secretion of cytokines, arrest of vacuole maturation, and enhanced intracellular nitric oxide production, all which contribute to the death of *M*. *tuberculosis*. These events themselves do not lead to initiation of granuloma, but their failure to eliminate *M*. *tuberculosis* and terminate events initiated by *M*. *tuberculosis* to counter them does. An interesting study suggested that one of the earliest steps in granuloma formation is the translocation of the macrophage from airspace into the lung interstitium, requiring IL-1–mediated crosstalk between *M*. *tuberculosis*–infected alveolar macrophages (AMs) and alveolar epithelium, which increased epithelial permeability (72).

ESAT-6, mKatG, SOD A, and Mtb-hsp have all been suggested as antigenic triggers for sarcoidosis granuloma (73–75). Genetic material for *M*. *tuberculosis* (e.g., *M*. *tuberculosis* rRNA and *M*. *tuberculosis* RNA polymerase and the mycobacterial virulence factor, SOD A) has been extracted from human sarcoidosis tissues (76–79), and a meta-analysis of 31 case series and case control studies found that 231 of 874 patients with sarcoidosis had positive mycobacterial or non-TB mycobacterial genetic material (77). PBMCs and BAL cells from patients with sarcoidosis also showed greater Th1 response when cocultured with ESAT-6 and/or mKatG compared with healthy controls (78, 80, 81). These findings provide support for low-abundance *M*. *tuberculosis* antigens as a trigger for sarcoidosis granuloma. Clearly, *M*. *tuberculosis* is not the only trigger of sarcoidosis, and many different antigens have been suggested — by epidemiological associations (e.g., pine pollens), animal studies (*Propionibacterium acnes*), and in vitro and human studies (vimentin) (82, 83). Some of the *M*. *tuberculosis* antigens implicated could also originate from nontuberculous mycobacteria. For example, it is difficult to distinguish *M*. *tuberculosis* and nontuberculous mycobacterial KatG. Autoantigens (e.g., vimentin) are more likely in the case of solid-organ sarcoidosis, e.g., cardiac and neurosarcoidosis (84, 85).

## Immune cells involved in formation of granuloma

### Macrophages.

The main cellular constituents of granuloma are a heterogenous group of immune cells with diverse functions in homeostasis, infection, and disease. In the lungs, two major groups exist, defined by their location, transcriptional profile, and ontogeny (86). The first macrophages encountered by *M*. *tuberculosis* are resident, yolk sac–derived AMs. These have a tolerogenic transcriptomic and functional profile, and their main task is to maintain alveolar homeostasis (87). In infection and disease, monocyte-derived (or bone marrow–derived) macrophages (MDMs) become more relevant, as they arrive to replace the infected AMs (88, 89). Macrophages have also been described as M1-like (proinflammatory and LPS/IFN-γ induced) and M2-like (antiinflammatory, prorepair, and IL-4 induced), but these divisions, especially in humans, are likely to be less simplistic (90, 91).

AMs and MDMs respond differently upon recognition of *M*. *tuberculosis*. Infection of resident AMs (which are highly permissive to *M*. *tuberculosis*) results in a metabolic commitment to fatty acid oxidation, while infection of MDMs is associated with upregulation of the glycolytic pathways (92). Glycolysis is a crucial metabolic prerequisite for phagocytosis, reactive oxygen species, and production of proinflammatory cytokines (93–95) resulting in macrophages with greater M1-like features. A driver of this glycolytic shift is mTORC1, a master metabolic checkpoint kinase that integrates microenvironmental signals to regulate the metabolism and proliferation of cells (96, 97). Zebrafish studies using forward genetic screen identified *mtorc1* as an important early host resistance factor in TB (98). In increasing glycolytic metabolism, mTORC1 signaling also appears to protect macrophages from mitochondrial damage and mycobacterium-induced cell death. In TB, this means that macrophages can counter an early mycobacterial virulence mechanism simply by regulating energy metabolism, while the host antigen-specific adaptive immune response gathers pace (98).

In sarcoidosis, chronic upregulated mTORC1 signaling in macrophages is proposed as a major factor in persistence of granuloma (99). Linke et al. found that activation of mTORC1 in myeloid cells (via myeloid-specific deletion of *Tsc2*) initiated and maintained granuloma formation via increased macrophage proliferation and inhibition of apoptosis in vivo (99). The level of mTORC1 activation in sarcoidosis lung samples also correlated with progressive disease, reflecting new granuloma formation (99). Linke et al. also showed that proliferation of MDMs is critical to development of epithelioid granulomas. This required de novo expression of cyclin-dependent kinase 4 (CDK4), metabolic reprogramming toward increased glycolysis, and simultaneous inhibition of NF-κB signaling and apoptosis. Other studies showed that coculture of sarcoidosis PBMCs with purified protein *M*. *tuberculosis* derivative resulted in increased mTORC phosphorylation, resulting in granuloma-like aggregates in cell culture (100), while inhibition of mTORC signaling caused a significant reduction in granuloma formation. Independently, in sarcoidosis, *CDK4* was identified as a key contributor to granuloma formation in a GWAS (101).

In addition to M1-like macrophages in the granuloma, there are some reports of M2-like/antiinflammatory macrophages in TB and sarcoidosis tissue (102, 103). *M*. *tuberculosis* can also push differentiation to an M2-like macrophage by increasing arginase-1 and reducing nitric oxide production, thus increasing *M*. *tuberculosis* survival (104, 105). The driver for M2-like macrophages in sarcoidosis is less clear, though IL-13, found in some fibrotic lungs, is a candidate (102, 106).

### Monocytes.

Monocytes are precursors to macrophages and are recruited to the lungs along the CCL-2 gradient (107). Similar to macrophages and neutrophils, they can also act as a permissive focus for mycobacterial multiplication and survival. Monocytes are recruited to the lungs in the initial stage of *M*. *tuberculosis* infection (108). The inflammatory or CD14^+^CD16^+^ monocytes subset is increased in TB, but there is some evidence that they have an immunomodulatory role (109–111), with a predisposition to differentiate to M2-like macrophages. There is also a significant correlation between M2-like macrophages abundance in TB lungs and progression of disease (110).

Patients with sarcoidosis also have higher levels of CD14^+^CD16^+^ monocytes in their blood and BAL compared with healthy controls (112). In contrast to monocytes from patients with TB, these monocytes have an inflammatory profile, with higher basal levels of TNF-α and IL-6, and upregulated TLR2 expression (113). Their levels are also associated with a progressive disease course in sarcoidosis (112). There is evidence that increased TLR2 expression on monocytes is important in granuloma formation. TLR2 stimulation by serum amyloid A can regulate granuloma formation in sarcoidosis independent of any direct infection of monocytes (114). In the lungs, BAL cells from patients with sarcoidosis showed enhanced secretion of TNF-α and IL-6 when stimulated with a TLR2 ligand (19 kDa lipoprotein of *M*. *tuberculosis*) (115). Circulating monocytes from patients with sarcoidosis also showed upregulation of genes involved in phagocytosis and lysosomal pathways, whereas genes involved in proteasome degradation and ribosomal pathways were downregulated (116).

Therefore, there are functional differences in circulating monocytes derived from patients with sarcoidosis and patients with TB. One hypothesis is that the antiinflammatory profile observed in TB is secondary to direct *M*. *tuberculosis* infection of the monocytes (if these were in the lungs), whereas changes in monocytes are a primary feature of sarcoidosis, linked to genetic variation, and precede monocytes’ arrival in the lungs. Indeed, polymorphisms in *TLR2* loci have been shown in sarcoidosis and are also associated with disease course (18).

### Neutrophils.

Neutrophils are important constituents of the TB granuloma (114, 117, 118). Blocking neutrophil expression of S100A9 (one of a group of calcium-binding surface proteins) prevented the formation of organized epithelioid granuloma in a guinea pig model (119). S100A9 expressed by neutrophils in mycobacterial granulomas was found to amplify prostaglandin E2 production and macrophage polarization to an M2-like phenotype (120). Neutrophils can phagocytose mycobacteria and may subsequently die by apoptosis, undergoing efferocytosis by resident macrophages (121). Neutrophil presence is not a well-established phenomenon in lungs of patients with sarcoidosis, and they are not usually found around granuloma. However, several studies have shown a small increase in neutrophils in BAL of patients with sarcoidosis, though their origin from an associated nonsarcoidosis-related tracheobronchitis remains uncertain (122–124). Their contribution to granuloma formation is unknown, but those with higher neutrophil levels in BAL were more likely to require corticosteroids treatment and had relapsing disease (122, 123).

### T lymphocytes.

T cells play an integral role in the maturation of granulomas. In *M*. *tuberculosis* infection, optimal control of the organism requires antigen-specific CD4+ T cells. Loss of CD4^+^ T cells (as in AIDS-HIV) and ability to produce IFN-γ result in devastating disseminated TB infection (125–129). Additionally, without CD4 Th1 cells, *M*. *tuberculosis* can disseminate, despite preservation of the cellular integrity of the granuloma (130). IFN-γ–deficient mice develop lethal mycobacterial infection, even in the setting of a low-dose inoculum (131, 132). However, an often-cited anomaly is the relatively poor ability of lung-resident CD4^+^ T cells to produce IFN-γ (in contrast to circulating CD4^+^ T cells), which may protect from *M*. *tuberculosis*–induced pathology (133). In a murine infection model, Gern et al. showed that IFN-γ production by *M*. *tuberculosis*–specific CD4^+^ T cells is rapidly extinguished within the granuloma but not within unaffected lung regions, suggesting localized immunosuppression (134). TGF-β seems to contribute to poor IFN-γ secretion by CD4^+^ T cells, as blockade of TGF-β signaling in T cells resulted in improved IFN-γ production within granulomas, and lower bacterial burdens. This phenomenon is not observed in sarcoidosis. Instead, CD4 Th1 cells are highly active within sarcoidosis-affected lungs (as measured by BAL) (135) even though there is relative peripheral lymphopenia and anergy in circulating lymphocytes (136).

Another effector CD4^+^ T cell, Th17, has also been shown to be important in both TB and sarcoidosis. In TB, it was proposed that the primary role of IL-17 is to clear *M*. *tuberculosis* that has not been controlled by Th1 cells and macrophages. In early *M*. *tuberculosis* infection, the main source of IL-17 seems to be γδ T cells (137, 138), but Th17 cells do appear in the lungs as infection progresses (139). IL-17A is instrumental in granuloma formation and stability and recall of IFN-γ–producing CD4^+^ T cells to the lungs (140, 141). In IL-17A–knockout mice, *M*. *tuberculosis* infection is accompanied by granuloma that failed to mature (partially due to reduced intercellular adhesion molecules and LFA-1) and impaired protective response (140). Conversely, unrestrained IL-17 levels promote detrimental host responses in TB infection, mainly due to the accumulation of neutrophils in infected tissue (142). As the infection progresses, IL-17 production declines. Potential mechanisms include Th17 cell exhaustion with high expression of PD-1 and downregulation of the IL-23 receptor on Th17 cells and dysregulation of the STAT3 pathway (143), limiting Th17 cells’ ability to produce IL-17.

In contrast to TB, Th17 cells are the main producers of IL-17 in blood and lung of patients with sarcoidosis and likely have a pathogenic role (144). A large genetic association of SNPs of >19,000 individuals detected the IL-23R/Th17 pathway as a prominent factor in causation of sarcoidosis (145). Th17 cells (shown by flow cytometry staining surface markers CCR6 and CCR4) in mediastinal lymph nodes of sarcoidosis are increased (146). There is also immunohistochemical evidence of IL-17 and IL-23R expression in granulomas (135) and higher levels of ESAT-6–stimulated IL-17–producing CD4^+^ T cells in BAL (147). However, IL-17–producing CD4^+^ T cells were also found to be reduced in sarcoidosis BAL by Tøndell et al. (148), who used IL-17 and IFN-γ expression to identify these cells.

In sarcoidosis, a further subset of Th17 cells that produces both IFN-γ and IL-17 (in variable amounts) is found and termed variably Th17.1, Th1/Th17, ex-Th17, nonclassical Th17, or Th17* cells. These cells are increased in BAL and blood (148), and they may be more important than Th17 cells in their contribution to disease and granulomas, owing to their contribution to the IFN-γ pool in the lungs. Th17.1 cells were shown to correlate with radiological stage (148, 149) and progressive chronic disease (150). These Th17.1 cells are thought to differentiate from recruited Th17 cells under the influence of local inflammatory signals and cytokines, e.g., IL-23, and especially IL-12, which suppresses RORγt and IL-17 but enhances IFN-γ (140, 151). However, in a study in which Th17.1 cells were more definitively defined by T-bet and RORγt expression, Kaiser et al. found that they were also increased in Löfgren’s syndrome (152). Vukmirovic also identified a Th17 signature transcriptomically in BAL cells from patients with Löfgren’s syndrome (153). Overall, there is enough evidence to suggest a role for Th17 cells and IFN-γ–producing Th17 cells in sarcoidosis, but how they promote granuloma formation in sarcoidosis is unclear. Additionally, it is unclear whether IL-17 itself (rather than the IFN-γ produced by Th17.1) has a pathogenic role in sarcoidosis. The presence of Th17.1 cells is supported by single-cell transcriptomic studies by Krausgruber et al., as described below (154). Notably, Th17.1 cells do not appear to be regulated by Tregs (155), and their presence has not been widely reported in TB.

Th2 cell activity is less established in TB and sarcoidosis granulomas. A change in the Th1/Th2 balance could be involved in development of fibrosis in sarcoidosis granuloma, but the supporting evidence is not robust (156).

While much less abundant in granulomas, CD8^+^ T cells show interesting spatial localization within the TB granuloma environment and are required for optimal *M*. *tuberculosis* control (157, 158). In early stages of infection, CD8^+^ T cells are distributed in a circular formation on the outer layer of the lymphocytic core, and with chronic infection, they become more interspersed with CD4^+^ T cells throughout the granuloma (159).

### Tregs and innate lymphoid cells.

There is a large body of work around Tregs and control of TB infection in murine and NHP models and human samples (160). Many studies show elevated Tregs in blood and BAL of patients with TB compared with those with latent disease and healthy controls. However, it is unclear whether high levels of Tregs are a consequence of inflammation or a risk factor for TB development. As Tregs are closely linked to differentiation of Th17 cells, many have questioned if Tregs are involved in controlling the numbers of Th17 cells in TB and sarcoidosis granuloma. On its own, TGF-β induces expression of both FoxP3 and RORγt — transcription factors critical in development of Tregs and Th17, respectively. FoxP3 inhibits the function of RORγt (161) and constrains emergence of Th17 cells (162). However, in the presence of IL-6, TGF-β promotes CD4^+^ T cell differentiation to Th17 cells. Most work in animal models describes an increase in Treg levels as *M*. *tuberculosis* infection progresses (160).

In sarcoidosis, Treg perturbances tend toward functional rather than numerical anomalies. In a large tissue-based study (*n =* 69 patients), Taflin et al. showed that Tregs proliferate and accumulate within sarcoidosis granulomas, but neither circulating nor tissue Treg numbers correlated with disease severity (163). Furthermore, in an in vitro model using PBMCs cultured with BCG-coated beads, Treg depletion accelerated granuloma growth in healthy controls, but not in patients with active sarcoidosis, suggesting a resistance of sarcoidosis T cells or monocytes to Treg influence. Tregs in sarcoidosis granuloma appear terminally differentiated, with reduced expression of CTLA4 (164). They retain their antiproliferative activity, but they were unable to inhibit IFN-γ and TNF-α production from autologous T cells, compared with Tregs from healthy controls (165).

Overall, Th1, Th17, and Treg balance in granulomas could determine progression of disease and granulomas in sarcoidosis. The ratio of circulating Tregs/Th17 cells is inversely correlated with disease activity, decreasing in those who develop relapsing pulmonary sarcoidosis and returning to normal with treatment (166). The ratio of BAL Tregs/effector T cells was also significantly higher among patients who developed clinical remission versus chronic pulmonary disease on long-term follow-up (167).

Innate lymphoid cells, including NK cells and unconventional T cell populations (e.g., γδ T cells, MAIT cells, invariant NKT cells), are infrequent (apart from NK cells) but potent immune cells. In TB, apart from γδ T cells and NK cells, no major role has been identified for unconventional T cells in granuloma formation. γδ T cells recognize TB phosphoantigens (e.g., HMBPP) (168) and are expanded in the earliest phase of *M*. *tuberculosis* infection (169, 170). NK cells recognize mycobacterial cell wall components and stress ligands expressed on infected cells and can directly kill *M*. *tuberculosis*–infected cells (171, 172). They play a particularly important role in immune-compromised individuals, who lack the ability to mount a sufficient T cell response. In contrast, the roles of NK cells, invariant NKT cells, and γδ T cells in sarcoidosis are not well defined — γδ T cells were not found in BAL or biopsy specimens of patients with sarcoidosis (173, 174), and very few NK cells are found in sarcoid granulomas (175). CD1d-restricted invariant NKT cells are reduced in blood and BAL and absent in lymph nodes of patients with sarcoidosis (176).

### B cells.

In the periphery of the granuloma in TB, B cells form highly organized ectopic lymphoid structures with the molecular and immunological characteristics of germinal centers (177–179). Maglione et al. demonstrated that B cell–deficient mice lacked germinal centers in the lungs and showed abnormal granulomatous responses correlated with increased pulmonary pathology and burden of infection (180, 181). Without B cells, *M*. *tuberculosis* infection is accompanied by high levels of IL-17–mediated neutrophilic recruitment and more severe lung immunopathology (182).However, when compared with CD4 Tfh-like cells, B cells were relatively less important in controlling *M*. *tuberculosis* burden (183). B cells are not known to be a major component of the sarcoidosis granuloma, with one exception — lung sections, particularly those from HLA-DRB1*03^+^ patients with sarcoidosis, show vimentin-rich tertiary lymphoid structures with a corresponding increase in both IgG and IgA antivimentin antibody titers in the blood (85). B cells may also play a role in some sarcoidosis phenotypes such as severe neurologic or ocular sarcoidosis, given reports of therapeutic responses to rituximab in otherwise treatment-refractory disease. Cross-talk between B and T cells may be relevant but largely unexplored.

**Figure 1:**
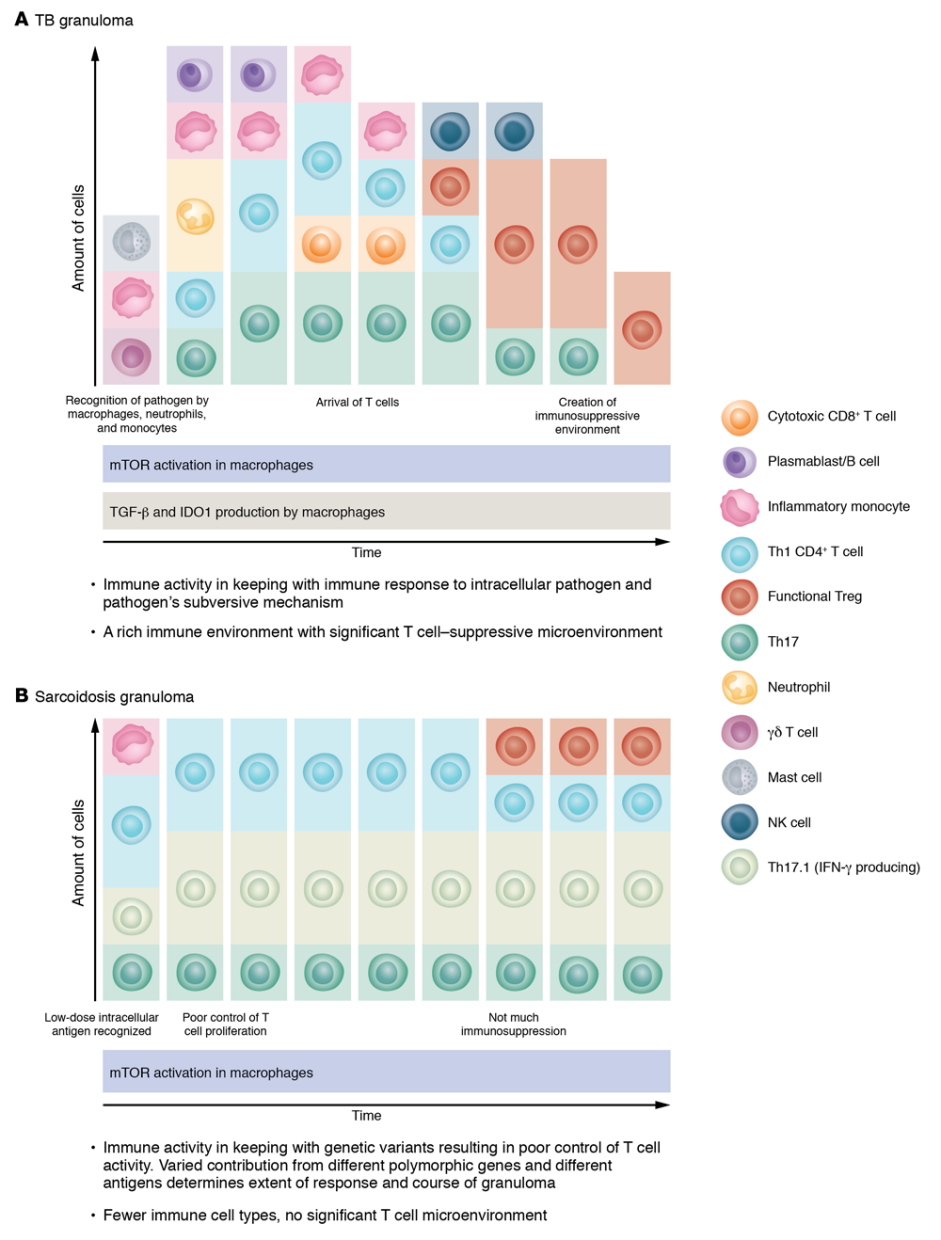
Schema of key immune features and immune cells (other than macrophages, MCGs, and epithelioid cells) involved or found in TB granuloma and sarcoidosis granuloma. Each panel proposes a timeline of involvement and relative abundance for each of the immune cells and major processes within (**A**) TB granuloma and (**B**) sarcoidosis granuloma. Each granuloma is thought to act as its own immune entity with its own trajectory.

These studies show key similarities and differences between sarcoidosis and TB in immune cells behavior around granulomas (summarized in Table 1 and Figure 1). Granuloma formation in TB is heavily driven by recognition of antigens from a specific pathogen (as expected) and is a product of a coordinated host immune response against an intracellular pathogen and its evasive strategies. In contrast, the immune response leading to and maintaining granuloma formation in sarcoidosis may be driven by diverse genetic variants.

## Single-cell analysis of granuloma in TB and sarcoidosis

Advances in single-cell omics technologies allow us an unbiased overall view of immune cells in tissue, with unprecedented detail and accuracy. Their in silico analyses can help generate hypotheses or confirm previous findings around intercellular circuitry. However, the number of patients involved in these analyses is typically low, which has to be borne in mind. Below, we analyze three key single-cell genomic profiling studies in granulomas, primarily in TB (154, 184, 185)

In the first study, McCaffrey et al. examined the single-cell immune and structural landscape of human lung granuloma in TB (*n =* 6 advanced granulomatous lesions [postmortem and therapeutic resections], *n =* 3 early [from diagnostic biopsies], and *n =* 4 from sites other than lung), and sarcoidosis (*n =* 10) (184). Lung sections were subjected to MIBI-TOF, a method that combines single-cell analysis and tissue location (186). Compared with transcriptomic studies, the markers available to analyze and annotate cells are limited to the 37 antibodies selected for the multiplex staining panel. The authors found multiple spatially discrete and immunologically distinct groups of cells or microenvironment within the TB granuloma. In the myeloid and MGC microenvironment, high expression of IDO1 and PD-L1 resembled tumor-associated macrophages in cancer and implied a highly suppressive microenvironment. The number of IDO1^+^ and PD-L1^+^ cells in these microenvironments correlated positively with Tregs, and discrete foci of immunosuppressive islands of cells comprising particularly high numbers of Tregs and IDO1^+^ and PD-L1^+^ cells were present within the core of the granuloma. Interestingly, despite this, TB granulomas lacked the expected compensatory increase in T cell checkpoint expression (increased PD-1) that is seen in cancer; PD-L1^+^ immune cells in granulomas far outnumbered PD-1^+^ immune cells. This could mean that myeloid immunosuppressive activity could occur independently of local signaling feedback by activated T cells, despite close proximity. This interpretation was supported further by observations of high TGF-β expression by myeloid cells, which corresponded to their PD-L1 and IDO1 expression levels. TGF-β was also expressed by lymphocytes but did not correspond with the immune-suppressive markers in the granuloma. These intriguing findings suggest that TB granuloma are a highly immunosuppressive environment, mediated by Tregs and macrophage-secreted TGF-β. Myeloid cells appear to drive the proliferation of Tregs at the expense of T cell activation, hence the lack of PD1-expressing T cells. To corroborate these findings, McCaffrey and coauthors examined the correlation between treatment response and expression in blood cells of patients with TB and observed that expression of PD-L1 inversely correlates with “cure” from TB (*n =* 71 patients).

In the sarcoidosis granuloma, McCaffrey et al. found fewer Tregs, CD8^+^ T cells, intermediate monocytes, and fibroblasts and markedly greater numbers of CD4^+^ T cells compared with the TB granuloma. Like in TB, there were very low numbers of PD-1^+^ T cells and high numbers of PD-L1^+^ myeloid cells. The most striking difference was the lack of IDO1 expression in sarcoidosis granuloma. Thus, the immunosuppressive microenvironment generated by high PD-L1 and IDO1 appears to be specific to TB granuloma. IDO1 is a metabolic enzyme that converts the essential amino acid tryptophan into immunoregulatory metabolites known as kynurenines (187). It is possible that IDO1 in TB was induced directly by *M*. *tuberculosis* (188) as an immune evasive mechanism. Some kynurenines can inhibit T cell proliferation by arresting the cell cycle and inducing apoptosis (189) while generating Tregs.

Altogether, although the numbers of the patient samples were small and the clinical variability is acknowledged to be high for both TB and sarcoidosis, the striking finding is presence of an immunosuppressive environment possibly generated by *M*. *tuberculosis* that is not seen in sarcoidosis.

In another high resolution single-cell spatial study, this time using single-cell whole transcriptomic methods (rather than the 30+-marker multiplex staining panel used by McCaffrey, et al.), Krausgruber, and colleagues examined the granulomas found in the skin of 12 patients with sarcoidosis and compared it with nongranulomatous skin in the same patient (154). This study supersedes previous studies in sarcoidosis granulomas due to the superior depth in annotation of cells. Angiotensin I–converting enzyme (ACE) and IFN-γ–activated genes were found to be upregulated in macrophages from granulomas, and the granuloma-associated macrophages were more activated, with a proinflammatory signature compared with nongranuloma macrophages in the skin.

The study also confirmed previous observations of increased mTORC1 signaling pathway genes, associating this increase with reduction in apoptosis genes and a glycolytic metabolic signature in these macrophages. *HIF1A* and other hypoxia-related genes were observed to be upregulated, reflecting an oxygen-low microenvironment in the granuloma. Within the granulomatous lesions, subclustering of macrophages revealed two further clusters, differentiated by expression of *ACE*, *CHIT1*, and *CSF1*, which seem to be found only in granulomas (as opposed to dermis and epidermis). CSF-1 is important for survival and differentiation of macrophages, and in this study, its expression correlated with the expression of Th17 differentiation-inducing genes *CCL20* and *IL23A*. Notably, unlike McCaffrey et al., Krausgruber et al. did not report TGF-β–expressing macrophages in sarcoidosis granulomas (154), though other studies have shown TGF-β staining in sarcoidosis granulomas (190). Thus, the granuloma microenvironment in sarcoidosis is highly inflammatory, driven by these IFN-γ–producing metabolically active macrophages.

The findings also definitively established a role for IFN-γ–producing Th17.1 cells in sarcoidosis granulomas. There was a striking presence of granuloma-associated T cells that expressed genes encoding transcription factors related to Th1 and Th17 (Tbet and RORC), CCR6, and IL-23R, which could fit with the Th17.1 subset described previously. These Th17.1 cells showed a chronically activated phenotype. Similarly, CD4^+^ T cells showed high *CTLA4* (and high *PD1*) gene expression, indicating chronic stimulation and exhaustion, supporting presence of poorly or insoluble antigen(s) that are not cleared, or that the host immune response failed to establish immunological tolerance. Additionally, the CD4^+^ T cells displayed upregulation of genes encoding cytokines and cytokine receptors (*CSF2*, *IFNG*, and *IL23R*) and chemokines and chemokine receptors (*CCL20*, *CCR6*, and *CXCR3*).

No neutrophils were mentioned, which could reflect difficulty in detecting these cells by single-cell transcriptomic methods. Another interesting finding is that fibroblasts around granulomas had features that could reprogram themselves to antigen-presenting cells. Most of these findings provide definitive support for the importance of metabolically reprogrammed macrophages, active IFN-γ–producing CD4^+^ T cells, and Th17.1 cells, which also contributed to IFN-γ excess in these structures. Krausgruber et al.’s study is limited by its exploration of skin granuloma rather than lung-based granuloma (154). Although the major histopathology features of the granuloma are similar, the surrounding tissue immune cells are different between the skin and lungs. For example, antigen-presenting capacity in the skin is dominated by the resident Langerhans cells, with lower numbers of monocytes. CD1a-restricted T cells, prominent in the skin, are not usually found in the lungs. These differences could impact the immunologic platform that drive granuloma formation in different organs.

In the third study, Gideon and colleagues used single-cell transcriptomic analysis to examine the correlation between immune and structural composition of granulomas and its mycobacterial burden. In contrast to McCaffrey’s studies where lesions were obtained from chronic disease, Gideon studied granulomas from low-dose infection of *M*. *tuberculosis* in NHPs (*n =* 4), at a relatively early point of disease (10 weeks after infection) (185). 26 granuloma lesions, excised with CT-guided detection, were digested and divided into high or low bacterial content and CFU and early or late granulomas by matching PET-CT features of the granuloma to early and late lesions on longitudinal PET-CT studies. Granulomas with features of early granuloma had greater numbers of mycobacteria. Early and high-burden *M*. *tuberculosis* granulomas were associated with presence of mast cells, plasma cells, fibroblasts, and endothelial cells, while late granulomas contained more abundant T and NK cells. Cytotoxic CD8^+^ T cells, Th17 cells, and Th1 T cells were significantly correlated (negatively) with mycobacterial burden in granulomas. Curiously, although the Th17 cluster was enriched for IFN-γ and TNF-α and transcription factors associated with Th17 differentiation (e.g., RORA and RORC), the IL-17 gene was not detected. These results align with previous findings showing that IL-17 is mainly produced by γδ T cells in early TB granuloma. Another possibility is that these are Th17.1 cells, which are also described in sarcoidosis (149). If this is the case, it will have to be assumed that the IFN-γ production from these Th17.1 cells outstrips that of IL-17. Several macrophage subsets were carefully subtyped using transcriptomic approaches, and one subset, the IFN-response gene– and IL-1β–enriched macrophage subset, was associated with high bacterial burden. There was no correlation between Tregs and mycobacterial burden in these NHP granulomas. This could be because Gideon’s NHPs received low-dose *M*. *tuberculosis* and had mild disease. Their study concluded that as granulomas progressed in development, NHPs developed the ability to kill *M*. *tuberculosis* in granulomas owing to the acquisition of adaptive immune cells within the structure, particularly, of CD8 cytotoxic cells, Th1 cells, and Th17 cells. Tregs may be a late feature of TB granulomas or a feature of more severe infection. A surprise was the distinct finding of mast cells, T2 immune environment, and fibroblasts in early TB granulomas.

## Conclusion

Recent studies on transcriptome of single cells in human disease and NHP models for TB and sarcoidosis have brought together decades of work in murine models, in vitro granuloma, and human tissue. Although sarcoidosis and TB granulomas have similar histopathologic features, immune events around granuloma formation in these two diseases have important differences. In TB, resident AMs are the earliest responders to the pathogen. Recognition of *M*. *tuberculosis* by these macrophages triggers a cascade of immune responses. The immune events in sarcoidosis are likely triggered by a variety of poorly degradable antigens, some recognized by T cells (given strong evidence for clonally expanded CD4^+^ T cells in some population, refs. 191–193) and others by macrophage and or monocytes, facilitated by an oversensitive monocyte sensing system (e.g., TLR2).

mTORC1 activation is important for both diseases and sustains longevity of the granuloma, but the cause of its activation is different for TB and sarcoidosis. In TB, the pathogen itself activates mTORC1, while in sarcoidosis it is more likely that related mutations in genes involved in autophagy and intracellular vesicular transport impact mTORC1 activity (194). In TB, CD4 and Th17 activity decreases as the disease progress, potentially due to adjustments in the local environment, where *M*. *tuberculosis* induces TGF-β production by the macrophages. In contrast, in sarcoidosis, Th1 and Th17.1 activity is present from the outset and shows little sign of abating. The ability to differentiate to Th17.1 cells, which is not observed in TB, could be important in maintenance of granuloma in sarcoidosis.

More work is required around tissue Th17 cells and their transcriptomic profile in different types of sarcoidosis subtypes, as studies have also shown that IL-17–producing T cells are reduced (or unchanged compared with healthy controls) in the lungs of some patients (148, 152, 195). What keeps macrophages in granulomas active in sarcoidosis also remains unknown, but a combination of a specific intracellular or persistent antigens in patients and genetic mutations that enhance mTORC1 signaling and promote poor control of T cell activity seem likely.

The absence of IDO1^+^ myeloid cells in sarcoidosis granulomas is their clearest difference from TB granulomas. In TB granuloma, the highly enriched IDO1 microenvironment inhibits T cell proliferation, which could explain the differences in CD4 Th1 and Th17 activity between the two conditions.

**Figure 2:**
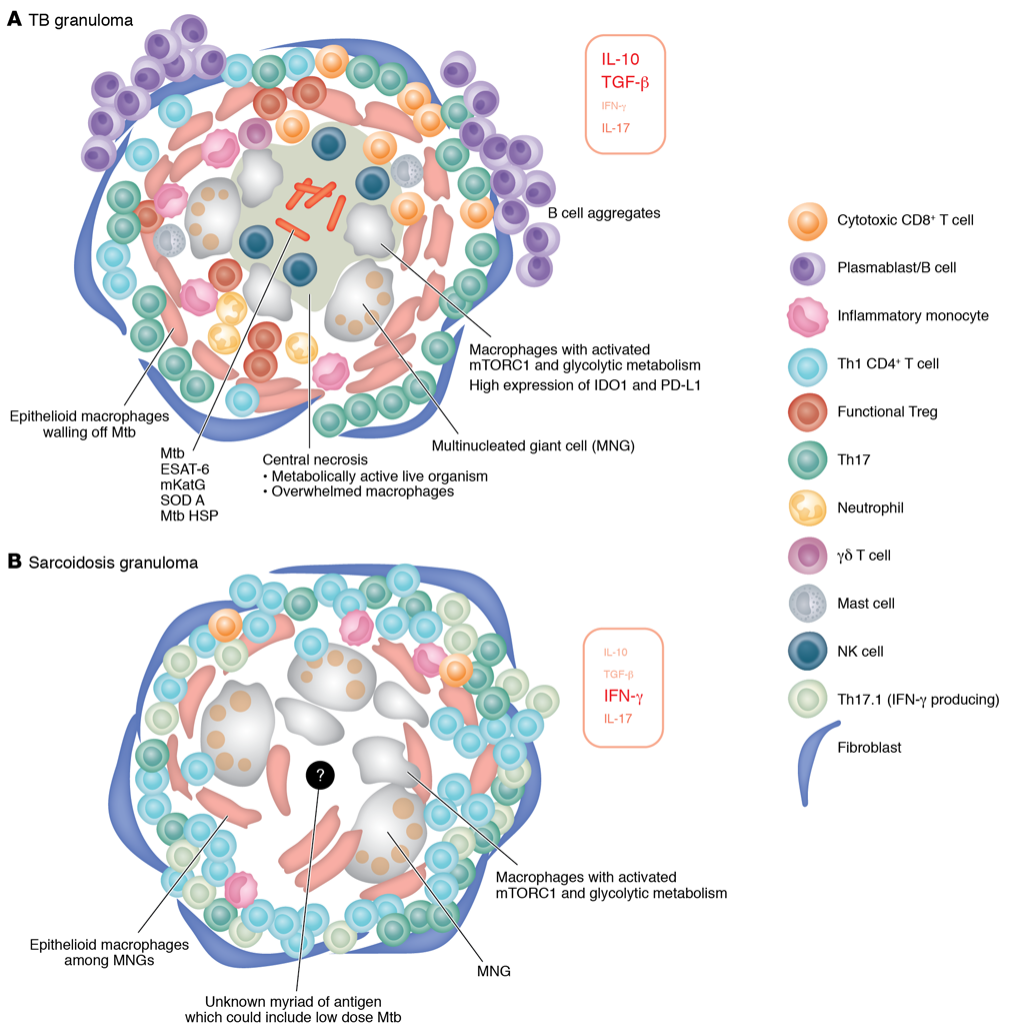
Diagram of TB granuloma and sarcoidosis granuloma. Each panel includes proposed key immune cells, key cytokines (the size of the font corresponds to prominence), and structural differences in (**A**) TB granuloma and (**B**) sarcoidosis granuloma. Note that granuloma is a dynamic structure. See Figure 1 for the corresponding changes during granuloma evolution, from initiation to resolution or chronicity. Mtb, *M*. *tuberculosis*.

Overall, TB granuloma formation and maintenance appears to be a study in the struggle between host and pathogen (Figure 2). There is a successful compromise whereby the host shuts down long-term damage once the burden of pathogens is reduced, with or without the aid of anti-TB treatment. This is a very different picture from sarcoidosis, where high level of T cell response, overactive monocytes, metabolic shifts, and poor T cell regulation is underpinned by multiple genetic variants and antigens.

## Supplementary Material

Supplemental table 1

## Figures and Tables

**Table 1:**
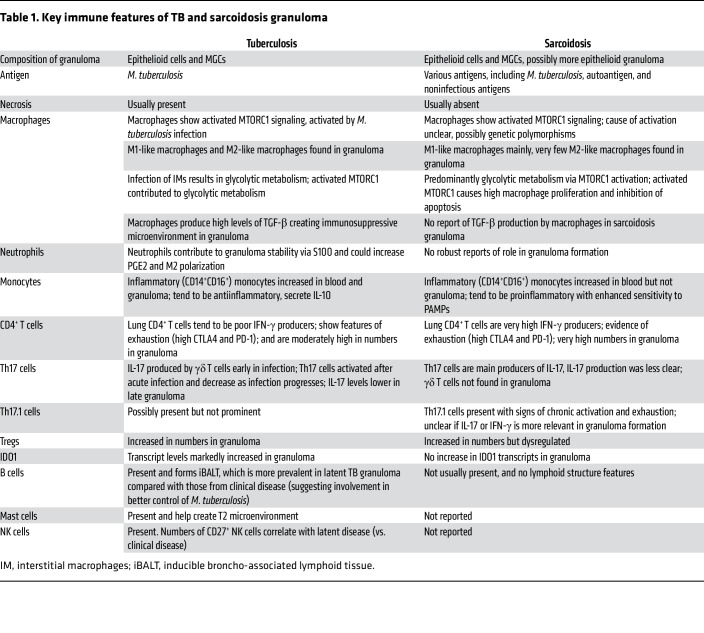
Key immune features of TB and sarcoidosis granuloma

## Version 1 01/02/2024

Electronic publication
