# Supplementary material for: Immune mechanisms of granuloma formation in sarcoidosis and tuberculosis
Source: J Clin Invest. 2024 Jan 2;134(1):e175264. doi: 10.1172/JCI175264 (PMC10760966; doi:10.1172/JCI175264)
Supplement: Supplemental table 1 [file jci-134-175264-s237.pdf]

Category	Gene polymorphism	Population	Association
**HLA gene locus**	HLA-DRB1 (*1101) and HLA-DPB1 (*0101) alleles	USA	Increased risk of sarcoidosis(8)
	HLA-DRB1 * 14, DRB1 * 15, and DQB1*0601	Japan, UK, Netherlands, Finnish	Chronic sarcoidosis [9, 10]
	HLA-DRB1*03 and DQB1*0201 alleles	Sweden	Increased risk of Lofgren (11) syndrome
	HLA-B * 51	Han Chinese	Increased risk of Lofgren (12) syndrome
	DRB1*0301	African American	Decreased risk of extra-pulmonary manifestations (13)
	DRB1*04 and DRB1*0302	Sweden	Increased risk of extra pulmonary and skin manifestations (14)
	HLA-DRB1 * 04	UK, Japan, European and African American cohorts	Sarcoidosis uveitis (9, 15)
	HLA-DQB1*0601	Japan	Cardiac sarcoidosis (16)
	HLA-DPA1/HLA-DPB1 HLA-DRB1	USA (New York fire department, 9/11 first responders)	Increased risk of sarcoidosis (17)
	HLA DRB1*14	Turkish	Increased risk of left ventricular diastolic dysfunction in cardiac sarcoidosis (18)
**T cell activation regulatory genes**	BTNL-2 rs2076530 – truncating splice site mutation BTNL2 promoter region (rs5007259)	Germany	T cell dysregulation, increased risk of sarcoidosis (19-21)

**Pattern recognition receptor encoding genes**	TLR 4 polymorphisms for the TLR4 gene (Asp299Gly and Thr399Ile)	Germany	Increased risk of chronic sarcoidosis (22)
	TLR-9 C-allele for T1237C	Germany	Increased risk of chronic sarcoidosis (22)
	TLR-2 AA-genotype at promotor location −16934	Netherlands	Increased in patients with chronic disease compared to patients with acute/self-remitting sarcoidosis (23)
	TLR -3 TLR3 polymorphism Leu412Phe (rs3775291)	Irish and American Caucasian	Significant association with persistent clinical disease in pulmonary sarcoidosis [(24)]
	TLR10-TLR1-TLR6 Absence of the common haplotype in the TLR10-TLR1-TLR6 gene cluster	Netherlands	Increases the risk of developing chronic disease in patients already affected by sarcoidosis (25)
	NOD2	France, UK	Sarcoid-related uveitis, and orofacial granulomatosis, including those associated with Blau syndrome (26, 27)
**Cytokines, chemokines and cytokine receptors**	TNF-α TNF-α -308 A/G and LT-α +252 A/G polymorphisms	Europe	Increased risk of sarcoidosis<br>−308 A > G, was shown to decrease the response to TNF inhibitors (28, 29)
	LTA Variant in LTA intron 1	Caucasian females (ACCESS study)	Associated with erythema nodosum in female Caucasian sarcoidosis patients (30)
	IL – 23R rs11465804 and rs11209026	Mixed ethnicity (Asian, Caucasian, African – American)	Sarcoidosis uveitis (31)
	IL12B	Germany	Central nervous system sarcoidosis<br>Increased risk of sarcoidosis (21)
	CCR5	Czech Republic	Progression of pulmonary sarcoidosis (32)

	CCL24	Japan	Increased risk of sarcoidosis (33)
**Cytoskeletal protein encoding genes**	RAB23 gene on chromosome 6p21 - non-synonymous SNP variant, rs10484410 (G207S)	Germany	Sarcoidosis uveitis (34)
	MAGI1	African American	Sarcoidosis uveitis (15)
**Autophagy**	XAF-1 intronic variant (rs6502976)	African American	Increased risk of sarcoidosis (35)
	mTORC1	France	Familial sarcoidosis (36)
**TGF beta genes**	TGFB3 4875 A<br>TGFB3 17369 C<br>TGFB2 59941 G	Netherlands	Over-represented in chronic fibrotic patients compared to those with acute/self-remitting and non-fibrotic chronic sarcoidosis (37)
	TGFB2 rs1891467	Germany	Protected from developing a chronic course (38)
	rs3917200	Germany	Fibrotic pulmonary sarcoidosis (38)
	TGF-β3 rs3917200*G	Greek	Associated with sarcoidosis (39)
**ANXA11**	ANXA11 Pathogenic SNP, rs1049550	German	Increased risk of sarcoidosis (40)
	209 SNPs in the ANXA11 rs61860052 rs4377299	African American	Associated with the risk of disease susceptibility and radiographic stage (41)
	ANXA11 rs1049550*A	Greek	Protective for sarcoidosis (39)
	non-coding SNP (rs61860052) of Annexin A11	African American	increased risk of sarcoidosis-associated uveitis and pulmonary fibrosis (42)
**Other**	NOTCH4 polymorphism in NOTCH4 (rs715299)	African American and European	Increased risk of sarcoidosis (36)
	TAP2	UK, Polish Slavonic	Increased risk of sarcoidosis (43)

	NF – κB	European	Increased risk of sarcoidosis (21)
	ZNF592	African American, European	Neurosarcoidosis (44)
	AADCL3	Finland	Resolving disease (45)
	STYXL1-SRRM3	Japan	Increased risk of sarcoidosis (33)
	PTGS2/COX2	USA (New York fire department, 9/11 first responders)	Increased risk of sarcoidosis (17)
	DBP rs4588 AA genotype	Serbia	Increased risk of sarcoidosis (46)
	GREM1 rs1919364	Netherlands	Fibrotic pulmonary sarcoidosis (47)

Table 1 Genetic association studies showing genes and loci associated with susceptibility and severity of sarcoidosis
